# Penetrating facial injury from angle grinder use: management and prevention

**DOI:** 10.1186/1746-160X-4-1

**Published:** 2008-01-23

**Authors:** Lachlan M Carter, Craig J Wales, Iain Varley, Martin R Telfer

**Affiliations:** 1 Specialist Registrar, Maxillofacial Surgery, Leeds Dental Institute, Clarendon Way, Leeds. LS2 9LU, UK; 2 Specialist Registrar, Maxillofacial Surgery, Regional Maxillofacial Unit, Southern General Hospital, 1345 Govan Road, Glasgow, G51 4TF, UK; 3 Senior House Officer, Maxillofacial Surgery, Pinderfields General Hospital, Aberford Road, Wakefield, WF1 4DG, UK; 4 Consultant Maxillofacial Surgeon, Maxillofacial Surgery, York District Hospital, Wigginton Road, York, YO31 8HE, UK

## Abstract

Injuries resulting from the use of angle grinders are numerous. The most common sites injured are the head and face. The high speed disc of angle grinders does not respect anatomical boundaries or structures and thus the injuries produced can be disfiguring, permanently disabling or even fatal. However, aesthetically pleasing results can be achieved with thorough debridement, resection of wound edges and careful layered functional closure after reduction and fixation of facial bone injuries. A series of penetrating facial wounds associated with angle grinder use are presented and the management and prevention of these injuries discussed.

## Background

Injuries resulting from the use of angle grinders are numerous. The most common sites injured are the head and face. The Royal Society for the Prevention of Accidents (RoSPA) Home and Leisure Accident Surveillance Systems (HASS/LASS) data collected from 2000 to 2002 showed that angle grinders were third in their top ten list of most dangerous tools, with an average of 5,400 injuries recorded yearly [1]. The increasing number of recorded angle grinder injuries during three consecutive years (2000 to 2002) reported in the HASS/LASS data is alarming. The vast majority of facial injuries are associated with foreign body penetration following shattering of the abrasive wheel. Open facial wounds are much less common, but can be very disfiguring, Table 1. We present a series of three penetrating facial wounds associated with angle grinder use.

**Table 1:** RoSPA – HASS/LASS data

Angle Grinder Injuries		
**Year**	**Total**	**Face**

**2000**	4,382	2,714
**2001**	4,712	2,945
**2002**	6,027	4,264

### Case 1

**Figure 1:**
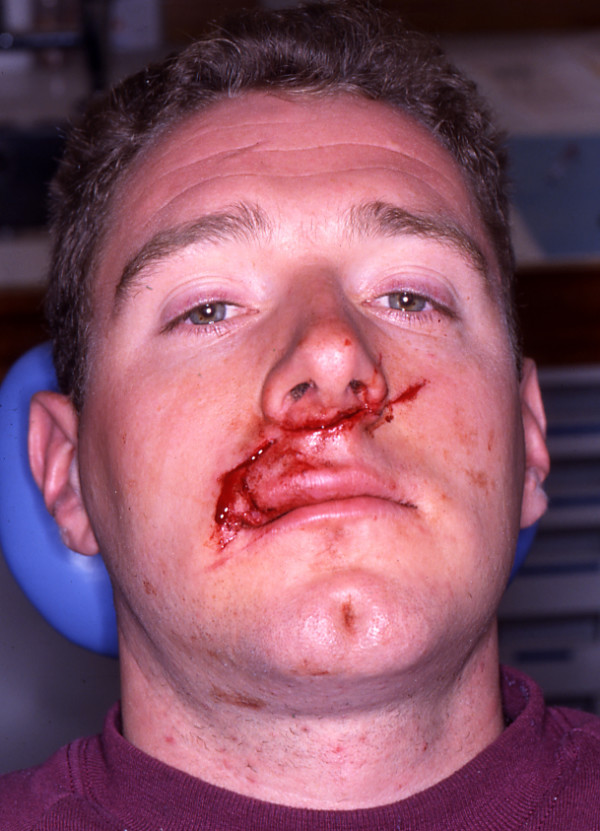
Case 1 – pre-operative appearance.

**Figure 2:**
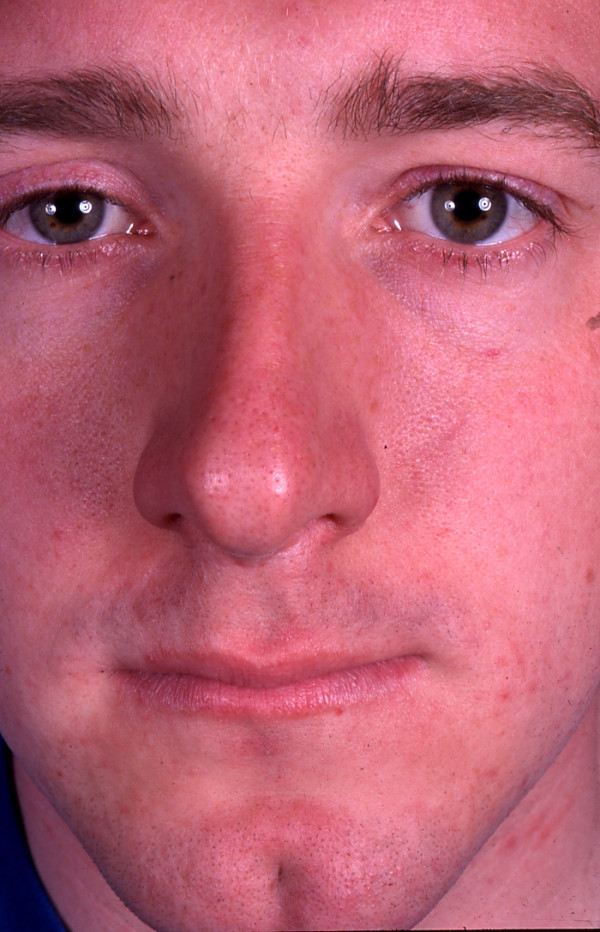
Case 1 – post operative appearance at 12 months.

Case 1 occurred when a left-handed, 26 year old male was injured as the blade of the angle grinder he was using shattered at high speed. He sustained deep wounds to his right upper lip, nasal base and left cheek, Fig 1. These wounds contained particulate matter from the abrasive wheel, requiring fastidious debridement. The wounds were debrided and closed in layers, under local anaesthetic. He recovered well post operatively and was discharged from clinic 12 months later, Fig 2.

### Case 2

**Figure 3:**
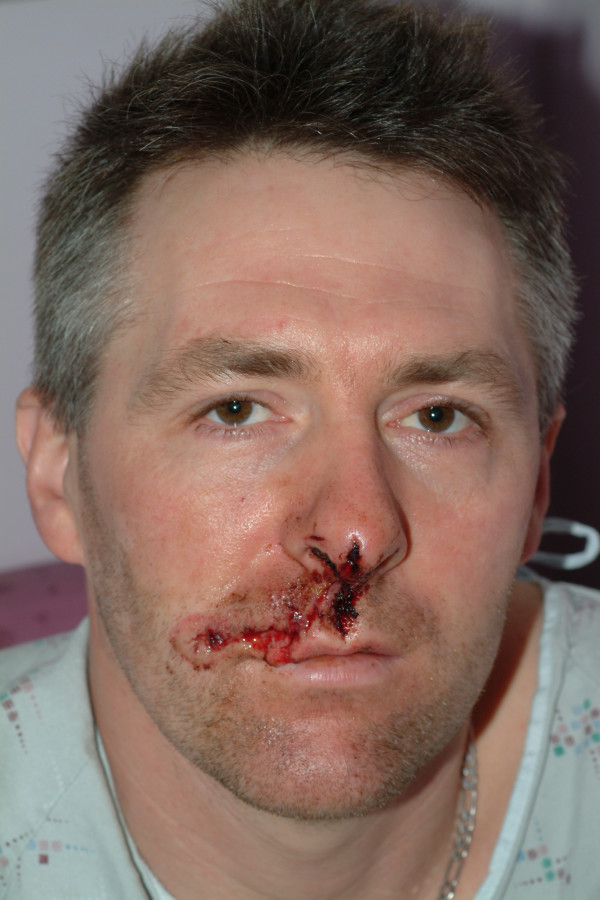
Case 2 – pre-operative appearance.

**Figure 4:**
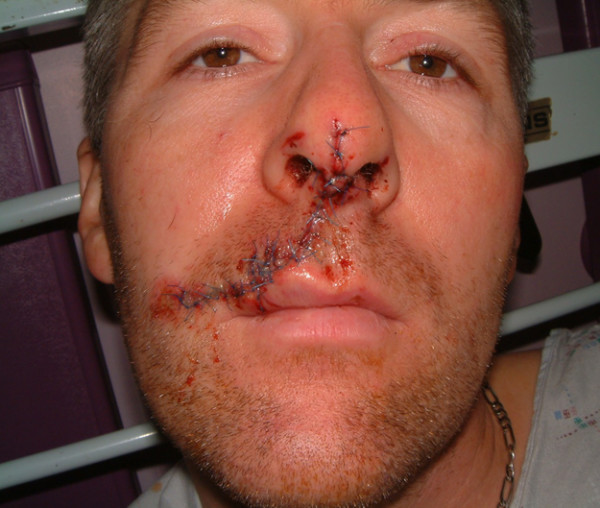
Case 2 – post operative appearance at 24 hours.

Case 2 occurred when a right-handed, 40 year old male was injured when the angle grinder he was using kicked back from the edge of a wooden plank. He sustained an open soft tissue wound involving the right upper lip, philtrum and nasal tip, Fig 3. Again the wounds were contaminated with material from the abrasive wheel and also the wooden plank. His wounds were debrided, carefully and closed in layers under general anaesthesia. He recovered well post operatively and was discharged from clinic 9 months later, Fig 4.

### Case 3

**Figure 5:**
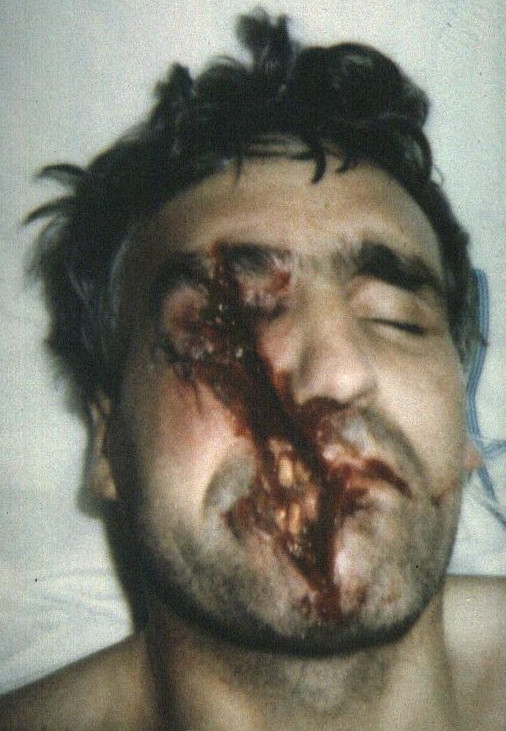
Case 3 – pre-operative appearance.

Case 3 occurred when a right-handed, 43 year old male was injured when the angle grinder he was using kicked up from the edge of a flag stone. The guard had been removed from the angle grinder by his neighbour and it was not replaced prior to its use. The patient sustained a linear open soft tissue wound on the right side of his face. The wound involved the chin, lips, cheek and supraorbital ridge. Unfortunately the right globe was also penetrated. The right mandibular parasymphysis, right maxilla and right supraorbital ridge sustained bony fractures, Fig 5. The wounds were debrided and closed in layers under general anaesthesia. The bony fractures were reduced and fixed with miniplates (parasymphysis and maxilla only). The right globe was enucleated and the final prosthesis fitted a few months later.

**Figure 6:**
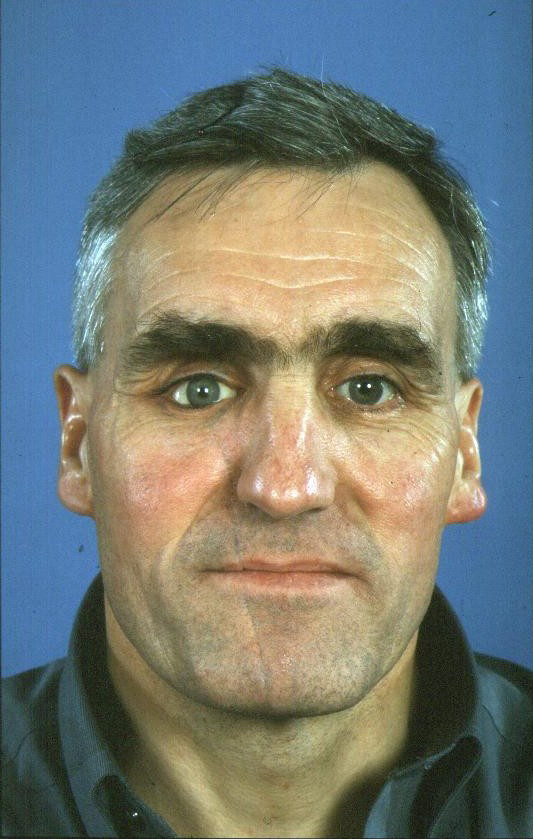
Case 3 – post operative appearance at 12 months.

The patient recovered well and was discharged from clinic 12 months post-injury, Fig 6.

In each of the cases the wounds were debrided with saline and wound edges heavily laden with particulate matter were excised. Oral mucosal and muscle layer closure was performed using Vicryl (polyglactin 910) resorbable sutures. Skin closure was preformed using non-resorbable monofilament interrupted sutures. Peri-operative intravenous Cefuroxime was administered for 24 hours followed by a seven day course of oral cephalosporin. Metronidazole was also administered in case 3. Chloramphenicol 1 percent ointment was applied to the skin wounds for seven days post-operatively. Wound review was performed at one, three and six weeks then at three, six and nine or twelve months.

## Discussion

Angle grinders are used around the world in large numbers to cut stone, metal and concrete [2]. They are also used to grind pre-welded joints and remove unwanted fragments of metal or ceramics. The discs themselves rotate between 6000 and 15000 revolutions per minute, depending on the machine type and the disc diameter used. As well as facial injuries, the main injuries are to the upper limbs and, less commonly, the lower trunk [1].

The morphology of the wounds sustained using angle grinders tend to follow the shape of the cutting disc; most often curvilinear but may vary slightly depending on the angle of skin entry. Tissue loss is a common feature. The volume of tissue loss is directly dependent on the size of the disc used. Finding fragments of disc and the material being cut in the wound is pathognomic of angle grinder injuries [3]. Therefore thorough debridement of contaminated wounds and excision of ragged edges is vital to optimal healing.

Injuries occur for a number of reasons. Firstly the wheel itself may kick back from the surface it is cutting. This will send the rotating disc toward the operator, parallel to the axis at which it is being used. Hence the face is most often at risk of a penetrating wound when looking down along the axis of the cuts being made [4]. This feature is present in all of the cases reported as all exhibit oblique/parasagittal lacerations parallel to the cutting axis. This risk is increased markedly if the guard has been removed as highlighted in case 3.

The other main reason for injury is the use of the wrong size/type of disc or a worn/chipped disc. This will increase the likelihood of excessive vibration and of the disc shattering. This usually results in foreign body type injuries. A thorough secondary survey should be performed in the situation of a shattered disc as several anatomical sites may be affected. In particular perineal or scrotal injuries occur if the operator straddles the object being cut and can be missed [2]. Overhead use of angle grinders has been associated with fatal intracranial injury and should be avoided [5]. A number of articles have been published to warn of these specific dangers [6,7]. In order to reduce the risks of injury there are general guidelines about the use of power tools such as checking they are maintained and on the use of protective clothing [7]. Specific guidance on the use of angle grinders is shown in Table 2.

**Table 2:** Safe use of angle grinders

Safe use of angle grinders	
**Safety measure**	**Advantage**

Use the correct disc size and replace the disc when wear is obvious or the disc is chipped	Reduces the risk of a foreign body injury as a result of disc disintegration
Stop using if vibration is very apparent	

Do not remove the guard unless for maintenance	Increases personal protection from direct and foreign body injury
Never use an angle grinder overhead	
Stand perpendicular to the plane of the cutting wheel, i.e. cut in a para-coronal plane to reduce the risk of kick back towards the sagittal plane of the body	
Always wear appropriate personal protective equipment/clothing (gloves, goggles, and hard-hat preferably with face shield)	

The cases presented illustrate that the high speed disc of angle grinders does not respect anatomical boundaries or structures. Aesthetically pleasing wound closure can be achieved with thorough debridement, resection of wound edges and careful layered functional closure after reduction and fixation of facial bone injuries. However the injuries produced can often be disfiguring, permanently disabling or even fatal and are mostly preventable. We suggest that before using such a power tool that both manufacturer's guidance and national guidelines should be consulted.

## Competing interests

The authors have no financial and personal relationships with other people, or organisations, that could inappropriately influence (bias) their work, all within 3 years of beginning the work submitted.

## Authors' contributions

LMC, CJW and IV prepared the case reports. LC and CW drafted the manuscript. MRT conceived the paper and coordinated the case report preparation. All authors read and approved the final manuscript.
